# Adherence to metformin in adults with type 2 diabetes: a combined method approach

**DOI:** 10.1186/s40545-022-00457-5

**Published:** 2022-10-12

**Authors:** Nadia Farhanah Syafhan, Rosemary Donnelly, Roy Harper, Janet Harding, Ciara Mulligan, Anita Hogg, Michael Scott, Glenda Fleming, Claire Scullin, Ahmed F. Hawwa, Gaoyun Chen, Carole Parsons, James C. McElnay

**Affiliations:** 1 Clinical and Practice Research Group, School of Pharmacy, Queen’s University Belfast, Belfast, UK; 2 Department of Clinical Pharmacy, Faculty of Pharmacy, Universitas Indonesia, Depok, Indonesia; 3 Ulster Hospital, South Eastern Health and Social Care Trust, Belfast, UK; 4 Medicines Optimisation Innovation Centre, Northern Health Social Care Trust, Antrim, Northern Ireland UK; 5 School of Biological Sciences. Faculty of Biology, Medicine and Health, University of Manchester, Manchester, UK

**Keywords:** Metformin, Adherence, Type 2 diabetes, Combined method approach

## Abstract

**Background:**

Medication adherence, one of the most important aspects in the process of optimal medicines use, is unfortunately still a major challenge in modern healthcare, and further research is required into how adherence can be assessed and optimised. The aim of this study was to use a combined method approach of self-report and dried blood spot (DBS) sampling coupled with population pharmacokinetic (PopPK) modelling, to assess adherence to metformin in adult patients with type 2 diabetes. Further aims were to assess metformin exposure levels in patients, determine factors associated with non-adherence with prescribed metformin, and to explore the relationship between adherence and therapeutic outcomes.

**Methods:**

A combined method approach was used to evaluate metformin adherence in patients with type 2 diabetes who had been prescribed metformin for a minimum period of 6 months. Patients were recruited from consultant-led diabetic outpatient clinics at three hospitals in Northern Ireland, UK. Data collection involved self-reported questionnaires [Medication Adherence Report Scale (MARS), Beliefs about Medicines Questionnaire and Centre for Epidemiologic Studies Depression Scale], direct measurement of metformin concentration in DBS samples, and researcher-led patient interviews. The DBS sampling approach was coupled with population pharmacokinetic (PopPK) modelling, which took account of patient characteristics, metformin dosage and type of formulation prescribed (immediate or sustained release).

**Results:**

The proportion of patients considered to be adherent to their prescribed metformin, derived from self-reported MARS scores and metformin concentration in DBS samples, was 61.2% (74 out of 121 patients). The majority (*n* = 103, 85.1%) of recruited patients had metformin exposure levels that fell within the therapeutic range. However, 17 patients (14.1%) had low exposure to metformin and one patient (0.8%) had undetectable metformin level in their blood sample (non-exposure). Metformin self-administration and use of a purchased adherence pill box significantly increased the probability of a patient being classified as adherent based on logistic regression analysis. Both HbA1c and random glucose levels (representing poor glycaemic control) in the present research were, however, not statistically linked to non-adherence to metformin (*P* > 0.05).

**Conclusions:**

A significant proportion of participating patients were not fully adherent with their therapy. DBS sampling together with the use of a published PopPK model was a useful, novel, direct, objective approach to estimate levels of adherence in adult patients with type 2 diabetes (61.2%).

**Supplementary Information:**

The online version contains supplementary material available at 10.1186/s40545-022-00457-5.

## Background

Good medication adherence is one of the most important aspects of medicines optimisation and a critical element in the medicines use process. It is one of the expected outcomes of the effective delivery of medicines optimisation [1]. Patients with type 2 diabetes who cannot achieve glycaemic control through lifestyle modification (e.g. diet and exercise) require pharmacological treatment such as oral antidiabetic agent(s) (OAAs) and/or insulin treatment. These treatments are not only important for glycaemic control but also for preventing or delaying diabetes complications [2, 3]. Unfortunately, poor medication adherence remains an important issue in diabetes patients and is a key factor in the failure to achieve treatment goals. It is associated with poor glycaemic control, increased incidence of hospital admissions, long-term complications, increased mortality rates and increased healthcare costs [4–7]. A systematic review which included 27 studies has reported that type 2 diabetes medication adherence ranged between 38.5 and 93.1% [4].

The cause of poor adherence to diabetes medication is often multifactorial. Systematic reviews of therapy adherence in patients with type 2 diabetes reported that dose regimen complexity, polypharmacy, the need to use injectable medications, and associated side-effects (e.g. hypoglycaemia, weight gain, cardiovascular problems) can all influence adherence. Perceptions relating to treatment safety and efficacy (necessity belief and concerns about medication), depression, economic considerations and the relationship between the patient and healthcare provider have also been identified as factors which can influence medication adherence [4, 8–10]. A number of studies assessing adherence to diabetes medications in patients with type 2 diabetes have reported that metformin has the lowest adherence rates when compared with other OAAs [11–15]. Published pharmacoepidemiological studies, which have assessed the influence of metformin adherence on patient outcomes, have recorded adherence rates ranging between 22.0% and 88.6% [11–18]. The research to date has involved indirect assessments of adherence to metformin therapy, which can overestimate or underestimate true adherence. It is strongly recommended to use a number of different approaches to assess medication adherence, and to triangulate the results obtained. This multi-method approach increases the validity and reliability of adherence data [19, 20].

Quantification of medicines and/or metabolites in dried blood spot (DBS) samples is a new direct approach to adherence assessment [21]. This method utilises very small volumes of blood (typically 15 μL) usually taken from a finger prick. It is a more convenient way of blood sampling especially in children, older people and psychiatric patients. Alternatively, DBS samples can be obtained via an aliquot of venous blood taken for another purpose. Samples collected are spotted onto, and dried on, an absorbent card (Guthrie card). When dried, this card can be easily handled, transported to the laboratory, stored without refrigeration and analysed, typically using high-performance liquid chromatography (HPLC) [21–26]. A validated approach for the determination of metformin in DBS samples has been developed within our laboratory [22].

Population pharmacokinetics (PopPK) is the pharmacokinetic study of a specific drug within a target patient population. The aim of PopPK studies is to build a model that relates drug dosage and covariates to blood concentrations [27–29]. Typical examples of covariates studied include patient demographics (e.g. age, sex), physiological and pathological factors (e.g. organ function), environmental factors (e.g. diet, smoking) and drug interaction [28]. PopPK modelling mainly uses a non-linear mixed effects modelling approach [30]. Available PopPK models for a target population can be used to provide estimates of expected plasma concentrations and can be applied to assess adherence by comparing actual with expected blood concentrations [21, 31, 32].

It is important to monitor adherence to metformin to prevent unneeded dose increases or the prescription of additional or replacement diabetes medication for those who are poorly adherent [22].

The aim of the present study was to evaluate adherence to metformin in patients with type 2 diabetes attending outpatient clinics in the South Eastern Health & Social Care Trust, Northern Ireland, using a combined method approach i.e. self-report and dried blood spot (DBS) sampling coupled with population pharmacokinetic (PopPK) modelling. Further aims were to assess metformin exposure levels in patients, determine factors associated with non-adherence with prescribed metformin, and to explore the relationship between adherence and therapeutic outcomes.

## Methods

### Study patients and study sites

Patient recruitment for the study was carried out at consultant-led diabetic outpatient clinics at the Ulster, Ards and Bangor Hospitals within the South Eastern Health & Social Care Trust, Northern Ireland.

Patients who attended a diabetic outpatient clinic at a study site hospital and who were aged ≥ 18 years, diagnosed with type 2 diabetes mellitus and prescribed metformin for a minimum period of 6 months were invited to join the study. Patients were excluded from the study if they were unable to give written informed consent, e.g. Alzheimer’s disease or learning disability, unable to communicate/complete study paperwork in English.

Patients interested in the study were given a proposed date for attending a research session at the hospital or if participants expressed a preference for the research session to be conducted on the same day during their visit to the hospital, this was accommodated.

### Sample size

Based on the assumption that adherence to medication in diabetes patients was approximately 50%, 196 subjects were needed to accurately estimate the percentage of medication adherence. This sample size provides 95% certainty that the estimate is within ± 7% of the exact population proportion [33]. We planned to recruit a target number of 220 patients to mitigate against missing data.

### Sampling method

Patients were recruited using convenience sampling, as described above, taking account of the study inclusion and exclusion criteria.

### Administration of self-completed questionnaires and collection of dried blood spot (DBS) samples

The Medication Adherence Report Scale (MARS) [34], the Beliefs about Medicines Questionnaire (BMQ) [35] and The Centre for Epidemiologic Studies Depression Scale (CES-D) [36] were given by the researcher (NFS) to each patient during a research visit to the hospital, and they were asked to complete these. The total MARS score ranges from 5 to 25. Higher scores represent better adherence levels. In this study, a cut‐off point of 90% [37] was used and the patients who had total scores ≥ 23 were considered adherent. The total BMQ scores of the necessity scale and the concern scale range from 5 to 25. Higher scores for the necessity scale are associated with greater patient perceived need for their medication to control their disease and maintain their health. On the other hand, higher scores for the concern scale are associated with higher patient concerns about harmful or adverse effects that are possible from long-term use of the medicine in question and worries regarding becoming dependent on their medications [35]. The CES-D was used as a tool for screening symptoms of depressed mood which can contribute to medication non-adherence. The CES-D score ranges from 0 to 60, with higher scores associated with greater depressive symptoms. A score of 16 or greater indicates depressed mood [36].

Having completed the questionnaires, a finger prick blood sample (automatic, disposable lancet) was taken by the researcher (NFS) from each patient and used to spot a Guthrie card with 15-uL aliquot using a disposable pipette. Date and time of collection were recorded for each sampling time. The blood spot samples were dried overnight in the dark at room temperature before storage at − 80 °C prior to analysis. As part of the process for the determination of metformin in DBS samples, haematocrit (Hct) values in the finger prick samples were measured during the research visit using Hemo_Control®.

### Quantification of metformin in DBS samples

The DBS samples were analysed for content of metformin using HPLC with UV detection, based on a validated method developed in house [22]. The method utilised 8‐mm disks (incorporating the full 15‐μL sample) punched from the Guthrie cards. Preparation of the DBS samples involved extraction using 1 mL of methanol. After centrifugation, the supernatant was transferred to a glass tube and dried under nitrogen at 40 °C for 30 min. This dried extract was then reconstituted with 120 μL of pure deionised water, vortexed for 5 min and then subjected to HPLC with UV detection (236 nm). The assay limit of quantification in DBS samples was 0.075 μg/mL. The intraday and interday accuracy and precision were within the limits recommended by International Council for Harmonisation (ICH) guidelines (± 15%).

### Population pharmacokinetic (PopPK) modelling

Prediction of metformin plasma concentrations associated with full adherence was based on a PopPK model developed by Bardin et al. [38]. To determine whether the patients were adherent, a computer simulation method was utilised to estimate the 95% interval of predicted plasma concentrations for metformin at the time of sampling relative to time of dose administration (*n* = 1000 sets of simulations using the non-linear mixed effect modelling software package, NONMEM version 7.4.4; Icon Development Solutions, Ellicott City, MD, USA).

Literature values of PopPK parameters for metformin were employed [38, 39]. In addition, significant covariates reported to influence PK parameters (e.g. age, lean body weight, daily dose and serum creatinine) were incorporated into the simulation models. For each patient, the measured DBS concentrations were transformed to corresponding plasma concentrations based on each individual patient's haematocrit level.

Patients with an undetectable metformin concentration or a calculated plasma concentration that fell outside the 95% confidence interval of the predicted full adherence plasma level [based on daily dose, time of last dose and time of blood spot collection] as determined by PopPK modelling [38], were classified as non-adherent. Patients were considered adherent if their metformin equivalent plasma concentration fell within the calculated 95% prediction intervals (2.5th and 97.5th percentiles) [21].

Metformin equivalent plasma concentrations were also used to assess the metformin exposure level by relating this value to the therapeutic range for metformin in plasma. If metformin plasma concentration was lower or higher than the metformin therapeutic range (1–5 μg/mL) [40–42], this was categorised as low or high exposure, respectively. Metformin levels below the limit of quantification in DBS samples were categorised as non-exposure.

### Collection of routine patient data

Demographic data (age, weight, height, sex and ethnic origin), medical history, current medications (and dosage regimes), and biochemical data (including serum creatinine, blood sugar levels and HbA1c levels) were recorded for each enrolled patient. These data were obtained from each patient’s medical records. All participating patients were interviewed by the researcher about their metformin and its management (e.g. dose regimes, how to remember to take medicine, any problems with medicine administration and potentially side-effects) using a study guide.

### Combined data analysis

Adherence for individual patients was determined based on combination of data derived from the concentration of metformin in DBS samples, together with the total score achieved from the MARS questionnaires. A patient was considered non-adherent if the MARS or the plasma level indicated that the patient was not following the prescribed dosage schedule [21].

Statistical analyses were carried out using SPSS version 25.0, SPSS Inc, USA. Descriptive statistics were used to describe patient demographics/characteristics and percentage of adherent and non-adherent patients. The Kappa coefficient (κ) was used to assess the extent of agreement between the adherence assessment methods [43].

Parametric and non-parametric tests were conducted as appropriate based on the normality of the data distribution. Adherent/non-adherent group differences were analysed using Chi-square analysis or Fisher’s exact test for categorical variables and t-test analysis for continuous variables. Spearman’s bivariate correlation was utilised to determine the relationship between adherence and continuous variables. Logistic regression analysis was also performed to determine the relative contribution of patient factors, including matters raised by patients during the patient interviews, to adherence with prescribed metformin.

## Results

**Fig. 1:**
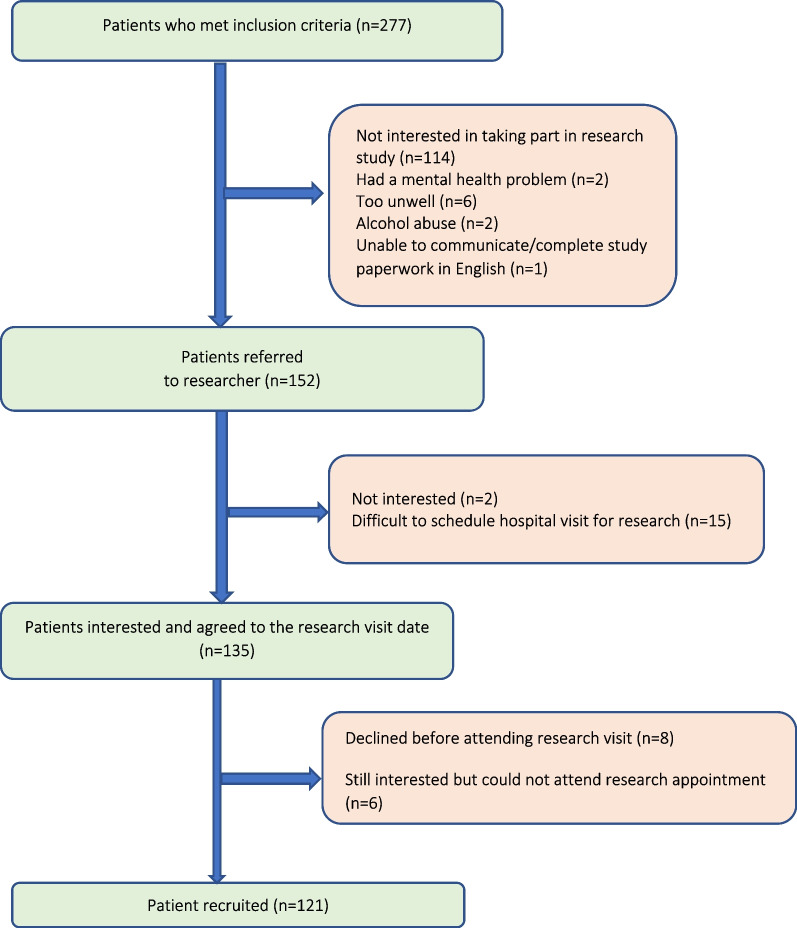
Flowchart of patient recruitment

A total of 277 patients met the inclusion criteria and 121 patients were recruited (43.6%). The total number of patients recruited was lower than anticipated since recruitment had to be terminated prematurely as a result of the cancellation of face-to-face outpatient clinics due to the COVID-19 pandemic. The pandemic also did not allow planned home DBS sampling to be implemented. Patient recruitment flow is presented in Fig. 1. A considerable number of patients (*n* = 125) were not referred by the clinicians to the researcher.

### Patient characteristics

Characteristics of the 121 patients recruited are presented in Table 1.

**Table 1:** Patient characteristics

Variable	Total
	*N* = 121
Duration of diabetes: [mean years ± SD]	14.7 ± 6.4
Age: [mean years ± SD]	± 9.7
18–65 years [n (%)]	45 (37.2)
> 65 years [n (%)]	76 (62.8)
Sex	
Female [*n* (%)]	38 (31.4)
Male [*n* (%)]	83 (68.6)
BMI: [mean kg/m^2^ ± SD]	34.1 ± 7.5
Lean Body Weight: (mean kg ± SD)	61.7 ± 11.8
Other medical condition	
No [*n* (%)]	2 (1.7)
Yes [*n* (%)]	119 (98.3)
Number of other medical conditions: [median [IQR]]	7 [3–11]
Number of antidiabetic medicines (oral or injection) prescribed: [median [IQR]]	3 [3, 4]
1 [*n* (%)]	2 (1.7)
2 [*n* (%)]	22 (18.2)
3 [*n* (%)]	62 (51.2)
> 3 [*n* (%)]	35 (28.9)
Number of all medicines prescribed: [mean ± SD]	10.8 ± 4.4
≤ 5 [*n* (%)]	8 (6.6)
6–9 [*n* (%)]	44 (36.4)
≥ 10 [*n* (%)]	69 (57.0)
HbA1c: [mean mmol/mol ± SD]	67.9 ± 17.9
Random glucose level: [mean mmol/L ± SD]	9.9 ± 4.1
Metformin concentration in plasma: [median μg/mL [IQR]]	1.6 [1.2–2.2]
Serum creatinine level: [mean μmol/L ± SD]	91.0 ± 32.4
Haematocrit level: [mean % ± SD]	39.1 ± 5.2

BMI: body mass index

On average, recruited patients had been living with type 2 diabetes for approximately 15 years and took, on average, three antidiabetic medicines. Almost all (98.3%) patients had other medical conditions and the mean ± SD number of all prescribed medicines was 10.8 ± 4.4. The mean ± SD levels of study population HbA1c and random glucose levels were 67.9 ± 17.9 mmol/mol (8.4 ± 3.8%) and 9.9 ± 4.1 mmol/L, respectively.

### Metformin regimens

More than half (60.3%) of recruited patients took immediate-release metformin tablets. Patients generally took metformin twice a day (79.3%) at a dosage level of 2000 mg per day (85.1%) (Table 2). During the interviews, it was observed that approximately half (*n* = 62, 51.2%) of recruited patients were taking metformin before meals instead of after meals (recommended direction).

**Table 2:** Metformin regimes taken by recruited patients

Variable	Total
	*N* = 121
Dosage form	
Immediate release [*n* (%)]	73 (60.3)
Single-component [*n* (%)]	70 (95.9)
Fixed-dose combination [*n* (%)]	3 (4.1)
Sustained/modified release [*n* (%)]	48 (39.7)
Frequency of administration daily	
Once [*n* (%)]	20 (16.5)
Twice [*n* (%)]	96 (79.3)
Three times [*n* (%)]	3 (2.5)
Four times [*n* (%)]	2 (1.7)
Daily dose	
1000 mg [*n* (%)]	14 (11.6)
1500 mg [*n* (%)]	1 (0.8)
1700 mg [*n* (%)]	1 (0.8)
2000 mg [*n* (%)]	103 (85.1)
2550 mg [*n* (%)]	2 (1.7)

### Adherence based on self-reported MARS

**Fig. 2:**
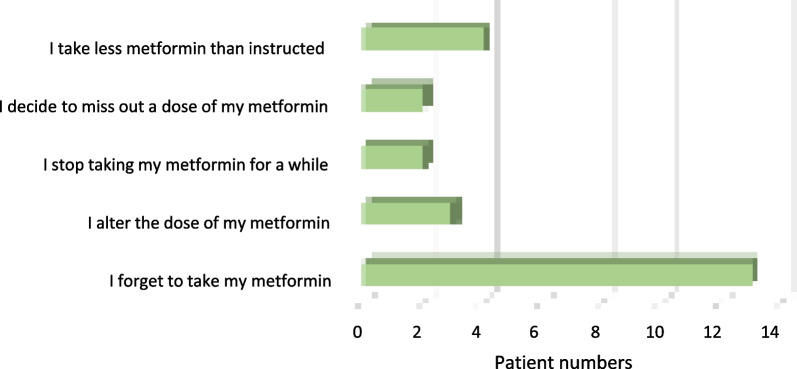
Number of patients who sometimes, often, or always engaged in non-adherent behaviours to metformin stated in the MARS questionnaire

All participating patients completed the MARS questionnaire. The questionnaire revealed that 110 patients (90.9%) scored ≥ 90% (23 points or greater) i.e. described themselves as adherent. Unintentional non-adherence was reported by 13 patients, who reported forgetting to take metformin at least sometimes, often, or always (Fig. 2) over a month.

### Beliefs about medicines and depressed mood

Three-quarters of patients (75.2%) had a BMQ necessity score that was above the scale midpoint, indicating that they had a strong belief that metformin was necessary for managing diabetes, while just under one-third of patients (31.4%) had concerns regarding the potential harmful effects of metformin prescribed for them. The mean ± SD total scores for the necessity and concern subscales were 17.7 ± 3.4 and 13.4 ± 3.6, respectively.

The majority of patients (*n* = 83, 68.6%), as measured by the CES-D questionnaire, scored < 16 indicating that they did not have a depressed mood or had a mild depressed mood. However, 18 patients (14.9%) had a moderate depressed mood, and 20 patients (16.5%) had a total score of > 23 which was indicative of severe depressed mood.

### Adherence based on metformin concentration in DBS samples

DBS samples were obtained from all recruited patients (*n* = 121) during their attendance at the hospital-based research session. According to the DBS sampling method, 75 patients (62.0%) were classified as adherent, whereas 46 patients (38.0%) were classified as non-adherent. From the non-adherent patient group, a total of 27 and 19 patients had metformin levels below and higher than the predicted plasma concentrations respectively. Examples of the PopPK model derived data for participating patients who were taking immediate-release and sustained-release formulations of metformin are shown in Additional file 1: Fig. S1 and Additional file 2: Fig. S2.

**Fig. 3:**
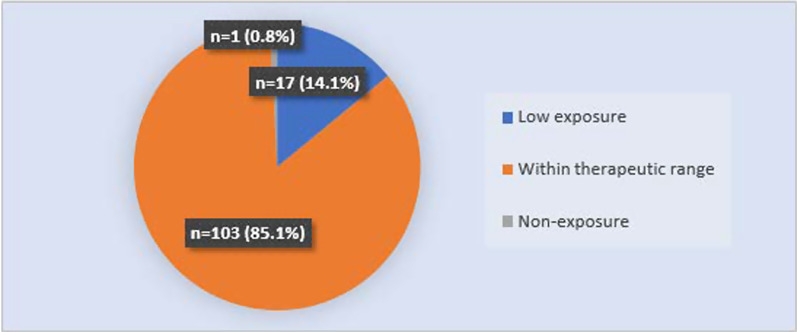
Level of metformin exposure in recruited patients

The majority (*n* = 103, 85.1%) of recruited patients had metformin exposure levels that fell within the therapeutic range. However, 17 patients (14.1%) had low exposure to metformin and one patient (0.8%) had metformin levels below the limit of quantification (non-exposure) (Fig. 3).

### Comparison of the different measures of adherence

As described earlier, a patient was classified as non-adherent if designated as such by one or both of the adherence measures (MARS and/or DBS approach) [21]. After recruited patients were categorised into adherent and non-adherent, the Kappa coefficient (κ) was utilised to assess the extent of agreement between adherence assessment methods (MARS and DBS samples). There was only a slight agreement between both measures since κ was 0.157.

**Fig. 4:**
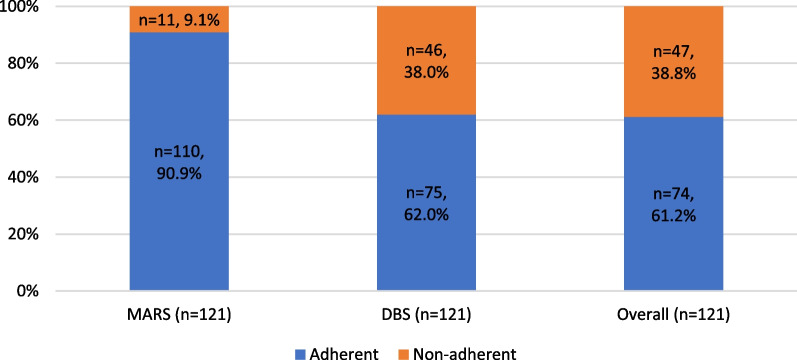
Comparison of results of metformin adherence classification of adult patients with type 2 diabetes using the different methods of assessment. MARS: medication adherence report scale; DBS: dried blood spot

The assessment method involving metformin concentration in blood captured higher percentages of non-adherence (38.0%) when compared with MARS (9.1%). Upon combining two adherence assessment methods, a total of 74 (61.2%) patients were considered adherent overall (Fig. 4).

### Linking non-adherence to patient outcomes

HbA1c and random glucose levels are deemed to be clear markers of poor clinical outcomes in patients with type 2 diabetes. Both HbA1c and random glucose levels representing poor glycaemic control in the present research were, however, not statistically linked to overall non-adherence to metformin (*P* > 0.05) (Table 3). These outcome parameters were also not statistically linked to non-adherence assessed by individual methods (MARS and DBS). In addition HbA1c and random glucose levels were not statistically linked to the level of exposure to metformin (Table 4).

**Table 3:** HbA1c and random glucose levels as outcomes for non-adherence

Variable	HbA1c (mean mmol/mol ± SD)	*P* value*	Random glucose level (mean mmol/L ± SD)	*P* value*
MARS				
Adherent (*n *= 110 [90.9%])	68.0 ± 17.7	0.787	10.0 ± 4.2	0.430
Non-adherent (*n *= 11 [9.1%])	66.5 ± 20.1		9.1 ± 3.6	
DBS				
Adherent (*n *= 75 [62.0%])	66.3 ± 17.6	0.215	9.5 ± 3.6	0.181
Non-adherent (46 [38.0%])	70.4 ± 18.2		10.6 ± 4.8	
Overall (MARS and DBS)				
Adherent (*n *= 74 [61.2%])	67.1 ± 18.2	0.554	9.8 ± 3.7	0.591
Non-adherent (*n *= 47 [38.8%])	69.1 ± 17.5		10.2 ± 4.8	

^*^t-test analysis

MARS: Medication Report Adherence Scale; DBS: dried blood spot; MRA: medication refill adherence

**Table 4:** HbA1c and random glucose levels as outcomes for exposure

Variable	HbA1c (mean mmol/mol ± SD)	*P* value*	Random Glucose Level (mean mmol/L ± SD)	*P* value*
Within therapeutic range (*n *= 103 [85.1%])	67.5 ± 17.9	0.612	9.8 ± 4.1	0.355
Low and non-exposure (*n* = 18 [14.9%])	69.8 ± 18.4		10.8 ± 4.4	

^*^t-test analysis

### Factors that influence adherence

Univariate analysis was performed for available variables in patients who were overall adherent and non-adherent to metformin (Table 5). Two variables were significantly associated with adherence to metformin (*P* < 0.05) using univariate analysis. These factors were (i) metformin self-administration (i.e. not being dependant on others to help with medication administration) and (ii) patient utilising a purchased adherence pill box. Logistic regression analysis indicated that both these factors were significantly and independently associated with adherence. Metformin self-administration and use of a purchased adherence pill box increased the probability of a patient being classified as adherent by 7.5 and 2.8 times, respectively (Table 6).

**Table 5:** Univariate analysis of possible factors affecting metformin adherence

Variable	Adherent	Non-adherent	*P* value*
	*n* = 74 (61.2%)	*n* = 47 (38.8%)	
Duration of diabetes			
Mean years ± SD	14.7 ± 6.7	14.7 ± 6.0	0.989
Age			
Mean years ± SD	65.6 ± 8.6	66.5 ± 11.2	0.678
Sex			
Female [*n* (%)]	23 (60.5)	15 (39.5)	1.000
Male [*n* (%)]	51 (61.4)	32 (38.6)	
Presence of comorbidities			
Yes [*n* (%)]	72 (60.5)	47 (39.5)	0.521
No [*n* (%)]	2 (100)	0 (0.0)	
Metformin formulation			
Immediate release [*n* (%)]	43 (58.9)	30 (41.1)	0.663
Sustained release [*n* (%)]	31 (64.6)	17 (35.4)	
Frequency of metformin			
Once daily [*n* (%)]	17 (70.8)	7 (29.2)	0.394
More than once daily [*n* (%)]	57 (58.8)	40 (41.2)	
Number of metformin tablets daily			
1–2 tablets [n (%)]	24 (54.5)	20 (45.5)	0.350
≥ 3 tablets [n (%)]	50 (64.9)	27 (35.1)	
Number of prescribed medicines			
Mean ± SD	10.8 ± 4.4	10.9 ± 4.3	0.847
Metformin self-administration			
Yes [*n* (%)]	72 (64.3)	40 (35.7)	**0.027**
No [*n* (%)]	2 (22.2)	7 (77.8)	
Living alone independently			
Yes [*n* (%)]	16 (57.1)	12 (42.9)	0.783
No [*n* (%)]	58 (62.4)	35 (37.6)	
Purchased adherence pill box			
Yes [*n* (%)]	44 (72.1)	17 (27.9)	**0.021**
No [*n* (%)]	30 (50.0)	30 (50.0)	
Pharmacy adherence packs			
Yes [*n* (%)]	9 (47.4)	10 (52.6)	0.277
No [*n* (%)]	65 (63.7)	37 (36.3)	
Reminder in mobile phone			
Yes [*n* (%)]	3 (100.0)	0 (0.0)	0.281
No [*n* (%)]	71 (60.2)	47 (39.8)	
Ability to read metformin label			
Yes [*n* (%)]	68 (61.3)	43 (38.7)	1.000
No [*n* (%)]	6 (60.0)	4 (40.0)	
Ability to get metformin out of packaging			
Yes [*n* (%)]	69 (61.6)	43 (38.4)	0.734
No [n (%)]	5 (55.6)	4 (44.4)	
Problems taking metformin			
Yes [*n* (%)]	4 (100.0)	0 (0.0)	0.156
No [*n* (%)]	70 (59.8)	47 (40.2)	
Presence of side effect			
Yes	11 (64.7)	6 (35.3)	0.956
No	63 (60.6)	41 (39.4)	
BMQ			
Necessity: mean score ± SD	17.8 ± 3.4	17.6 ± 3.5	0.786
Concern: mean score ± SD	13.5 ± 3.6	13.3 ± 3.7	0.742
Necessity-concern differential: mean score ± SD	4.3 ± 4.1	4.3 ± 4.1	0.949
CES-D			
Median score of CES-D [IQR]	11.0 [5.0–19.25]	11.0 [5.0–17.0]	0.785

*Significant at value < 0.05

BMQ: Belief about medicines Questionnaire; CES-D: Centre for Epidemiologic Studies Depression Scale

**Table 6:** Factors independently linked to metformin adherence using logistic regression analysis

Independent variable	*B*	SE	Odds ratio	*P* value	95% CI
Metformin self-administration	2.013	0.850	7.482	0.018	1.413–39.614
Purchased adherence pill box	1.043	0.401	2.836	0.009	1.293–6.224

## Discussion

Since medication adherence is best assessed using a number of different approaches and triangulating the results obtained, the present study used a combination of approaches to increase the validity and reliability of the adherence data collected [19, 20]. It also demonstrated the utility of the finger prick DBS sampling approach to assess metformin adherence in adult patients.

Intraindividual and interindividual variability related to measured concentrations in blood can, however, be a limitation in the use of blood levels for adherence assessment [44]. In the present study, the application of PopPK models was used to minimise these uncertainties, and to increase the robustness of the overall approach. The PopPK of metformin in adults has been reported in previous research [38, 39, 45]. The PopPK model developed by Bardin et al. [38] was selected as the most suitable for use in the present study population, because the clearance model includes age, lean body weight and serum creatinine level as covariates—all of which are of particular relevance to the present population. This model was appropriate for immediate-release formulations and also works well for sustained-release formulations [39]. Concomitant use of some medications such as bictegravir, cimetidine, dolutegravir, guanfacine, mexiletine, pitolisant, ribociclib, telaprevir, topiramate and vandetanib [46] can increase metformin concentrations, but in this present research no patients were taking any of these medications.

The proportion of patients in this study who were adherent measured by the MARS (90.9%) was approximately the same as the proportion of adherent patients reported in a previous study of diabetes medication (89.5%) where the MARS score of 23 was used as the cut off to categorise patients as adherent [37]. Overestimation of the adherence rate may, however, result from patient reluctance and unwillingness to disclose their non-adherent behaviour or report not taking medicines as prescribed [47, 48].

In the present study, the proportion of patients who were classified as being adherent to metformin using the DBS approach was much lower than that reported using the MARS. Some of the patients were classified using the current DBS/PopPK approach as over-adherent, i.e. had higher blood levels than estimated by the PopPK modelling. This could indicate a shortcoming in the modelling or “white coat adherence” in which a patient may have taken an extra dose of metformin, i.e. exceeded the prescribed amount, prior to their visit to the research session [49–51].

Regarding the exposure to metformin, only one patient had undetectable metformin level in their blood sample (non-exposure). This indicated that the patient had discontinued metformin.

In this present research, the HbA1c levels of those patients classified as over-adherent were higher, although not statistically significant, (mean ± SD: 69.6 ± 21.1 mmol/mol) than in adherent patients (mean ± SD: 66.3 ± 17.6 mmol/mol), highlighting the possibility of patients taking more metformin than prescribed before coming to the clinic to mask previous non-adherence [49–51]. This has been reported by previous studies in patients with diabetes [52–54].

### Comparison of the different measures of adherence

The proportion of patients considered overall adherent to metformin, derived from the subjective (self-reported MARS) and objective (metformin concentration in DBS samples) measures, was 61.2%, which is within the internationally reported range of the proportion of patients adherent to metformin in a type 2 diabetes population [11–18, 55, 56]. A study assessing medication adherence using a combination of subjective (self-reported questionnaire) and objective (pill counts) measures in adult patients receiving OAAs, lipid lowering, antihypertensive and/or antiplatelet agents, reported that 81.3% of patients were classified as adherent based on self-reported questionnaire results, while only 35.4% of patients were classified as adherent based on pill counts [57].

### Linking non-adherence to clinical outcomes

In the present study, significant correlations (*P* < 0.05) were not found between adherence classification and HbA1c or random glucose concentrations. The association between adherence to diabetes medication and glycaemic control has been reported in many published studies [5, 7, 17, 58–60]. In the present study, although not statistically significant, non-adherent patients had slightly higher levels of HbA1c (mean ± SD: 69.1 ± 17.5 mmol/mol) compared with adherent patients (mean ± SD 67.1 ± 18.2 mmol/mol).

The level of metformin exposure was also not statistically linked to both HbA1c and random blood glucose levels. The mean levels of HbA1c in patients who were exposed to metformin within its therapeutic range were considered high (67.5 mmol/mol). However, the fact that almost all patients were prescribed more than one diabetes medication at the time of their engagement with the study and that patients may have had considerable variation in adherence with lifestyle measures recommended in diabetes management, add additional challenges to clinical outcome data interpretation. What is clear is that metformin adherence or exposure do not appear to be major drivers of glycaemic control in this patient population.

### Factors influencing adherence

Metformin self-administration and the patient reporting their use of a purchased adherence pill box were variables that independently (*P* < 0.05) predicted metformin adherence in the present study (logistic regression modelling). Metformin self-administration in the present research refers to patients themselves being responsible for the storage and administration of their own metformin, without any help from others. Positive associations between metformin self-administration and adherence to metformin could be linked to the longer duration of diabetes. The majority of patients who self-administered had been living with diabetes for a long duration indicating they had good knowledge about their metformin and its dose regimes. A large study assessing determinants of diabetes medication adherence reported that good medication adherence was associated with longer diabetes duration [61]. The Capability, Opportunity and Motivation (COM) B model of behaviour developed by Michie et al*.* [62] shows that to undertake medication-taking, individuals should have the physical and psychological capability as well motivation to do so; in particular, to adhere to the instructions related to their medication. Capability and motivation can be achieved through increasing knowledge and understanding.

The use of a purchased adherence pill box was linked with adherence in the present research. Systematic reviews have found evidence that pillboxes are an effective intervention to address medication adherence issues [63–65]. They can be an effective strategy to improve medication adherence as they assist patients to remember to take their medicines and simplify complicated medication regimens [66, 67].

The present study did not confirm the hypothesis that necessity belief about metformin was positively associated with metformin adherence, or that concerns about metformin was negatively associated with adherence (as measured by MARS or DBS sampling method individually or combined). Other studies, including systematic reviews on medication adherence in patients with type 2 diabetes, have reported that believing that medications are important or necessary was positively associated with medication adherence, while having concerns about the medication negatively impacted adherence [8, 68, 69]. The majority of patients (75.2%) in the present study had a BMQ necessity score above the scale midpoint, indicating that they had a strong belief that metformin was necessary for managing diabetes, while about only one-third of patients (31.4%) had concerns regarding the potential harmful effects of metformin prescribed for them. This indicates that medical team have done a good job in this regard.

Again, there was no significant association between the CES-D score and non-adherence to metformin. A number of previous studies have found that depression is a predictor for non-adherence to diabetes medication [4, 70–73]. Patients with diabetes have been found to be 1.4–3 times more likely to have depression compared to non-diabetic patients. The prevalence of major depression in patients with DM is mostly estimated around 12% (ranging from 8 to 18%) [73]. Compared with the latter study results, the number of patients in the present study who had a moderate-to-severe depressed mood as measured by the CES-D questionnaire (31.4%) was considered high.

### Limitations

The present study had a number of limitations. Firstly, the sample size was smaller than planned, due to the COVID-19 pandemic cutting short recruitment, thus reducing the statistical power of the study. The pandemic also did not allow planned home DBS sampling to be implemented. Furthermore, there is a risk of recall bias associated with the self-reported MARS scores, which may impact the high adherence revealed using this measure compared to that reported with DBS samples. Since adult patients with type 2 diabetes are usually prescribed a combination of hypoglycaemic agents, the clinical outcomes evaluated in the present study, i.e. HbA1c and random glucose concentrations could be associated with the use of other diabetes medications and of course adherence to the advice on diet and exercise provided to all type 2 diabetic patients. Adherence assessed by DBS sampling during research visit and the MARS over a month might not be reflective of cumulative HBA1C over the last 3 months.

## Conclusions

DBS sampling together with the use of a published PopPK model to estimate metformin concentrations was shown to be a useful and novel, direct, objective approach to estimate levels of adherence in adult patients with type 2 diabetes. Being classified as non-adherent to metformin did not significantly influence clinical outcomes such as HbA1c and random glucose level. Metformin self-administration and use of a purchased adherence pill box by a patient were associated with increased probability of a patient being classified as adherent based on logistic regression analysis. The results suggest that attention should be given to the education of patients receiving metformin, with involvement of a caregiver as needed, to ensure that the patient is capable of self-administration of medication and recommend use of an adherence pill box. Further investigations of adherence utilising the DBS method should be carried out for other diabetes medications.

## Supplementary Information

**Additional file 1: Fig. S1.** Example of simulated metformin concentrations derived from PopPK model for patient aged 76 years old with lean body weight 47 kg and serum creatinine level 124 µmol/L who was receiving an immediate-release formulation of metformin at a dose of 1 g twice daily.

**Additional file 2: Fig. S2.** Example of simulated metformin concentrations derived from PopPK model for patient aged 70 years old with lean body weight 49 kg and serum creatinine level 129 µmol/L who was receiving a sustained-release formulation of metformin at a dose of 1 g twice daily.

## Data Availability

The data used to support the findings of this study are included in this published article.
