# Supplementary figures and images for: Adherence to metformin in adults with type 2 diabetes: a combined method approach
Source: J Pharm Policy Pract. 2022 Oct 12;15:61. doi: 10.1186/s40545-022-00457-5 (PMC9554867; doi:10.1186/s40545-022-00457-5)

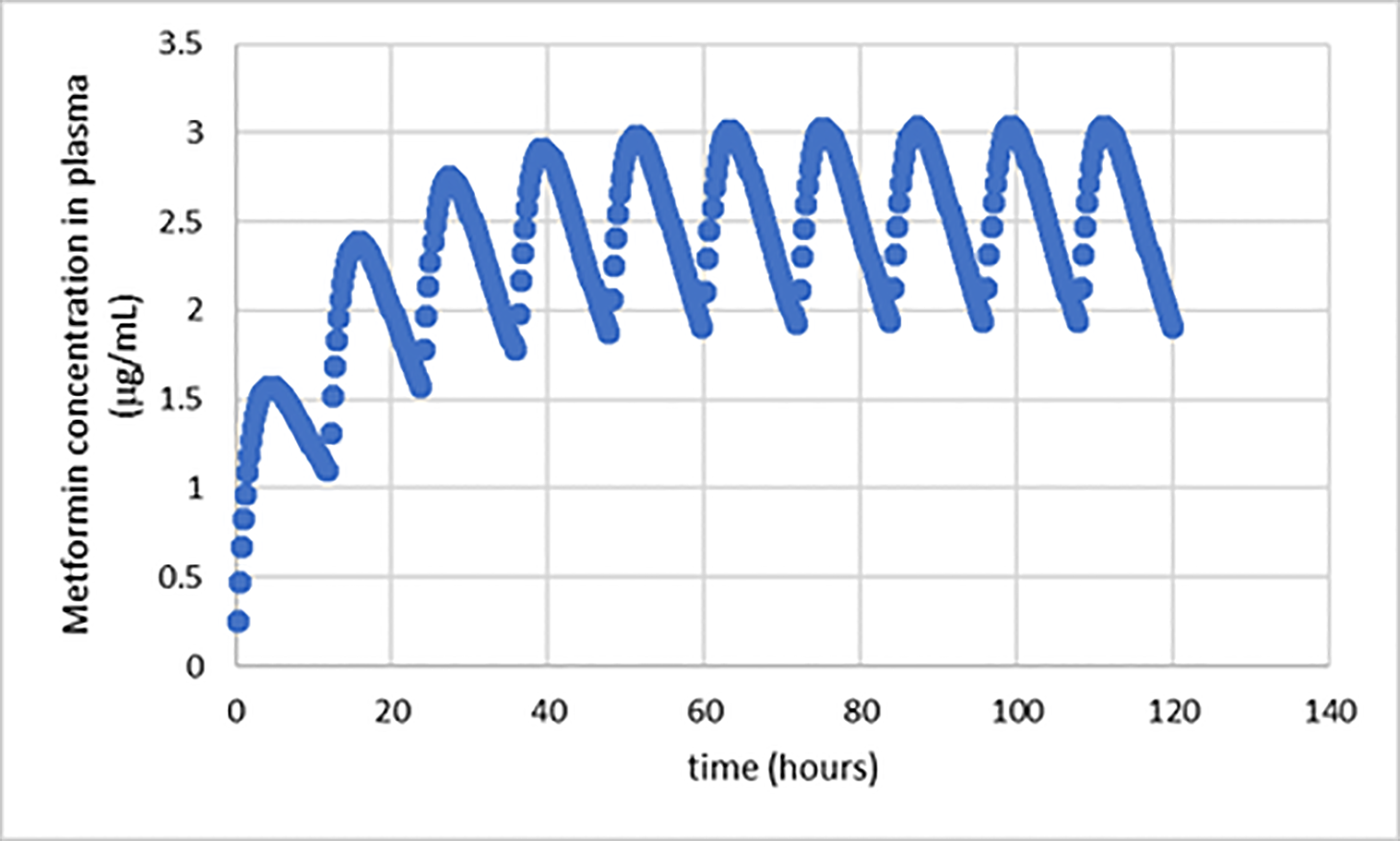

Supplement: Supplementary file 1 — Additional file 1: Fig. S1. Example of simulated metformin concentrations derived from PopPK model for patient aged 76 years old with lean body weight 47 kg and serum creatinine level 124 µmol/L who was receiving an immediate-release formulation of metformin at a dose of 1 g twice daily. [file 40545_2022_457_MOESM1_ESM.tif]

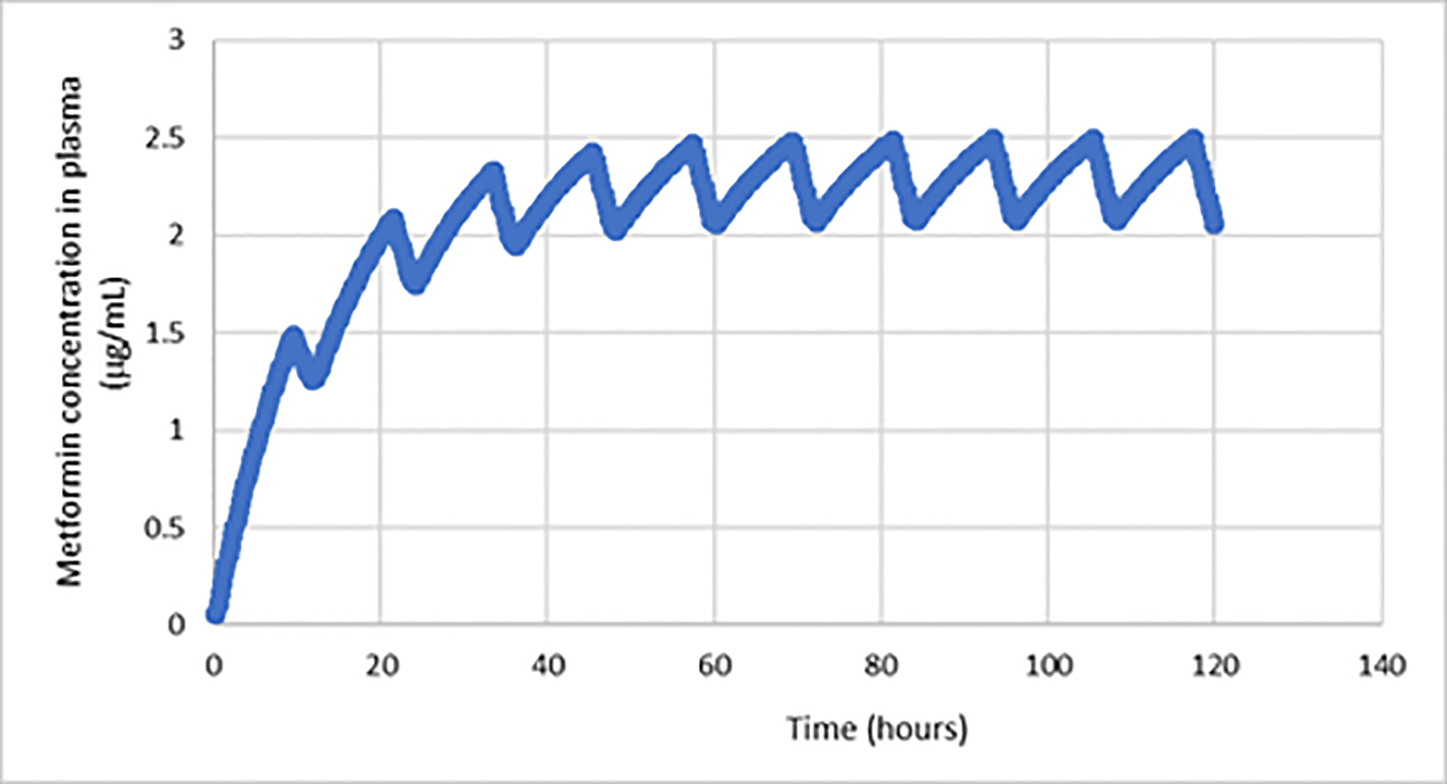

Supplement: Supplementary file 2 — Additional file 2: Fig. S2. Example of simulated metformin concentrations derived from PopPK model for patient aged 70 years old with lean body weight 49 kg and serum creatinine level 129 µmol/L who was receiving a sustained-release formulation of metformin at a dose of 1 g twice daily. [file 40545_2022_457_MOESM2_ESM.tif]
